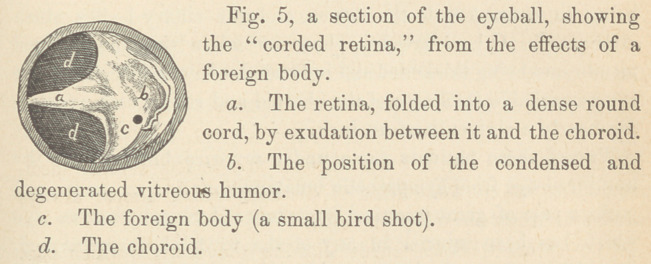# Mechanical Injuries of the Globe—with Numerous Pathological Specimens

**Published:** 1878-01

**Authors:** E. L. Holmes


### Mechanical Injuries of the Globe—With Numerous Pathological Specimens.

By Dr. E. L. Holmes.

*(Presented to the Chicago Medical Society.)*

Some weeks since, I placed before this society several specimens illustrating the effects of diseases, which almost always necessitate the removal of the globe.

I now present 28 specimens showing the changes which take place in the globe after certain injuries, and which render the removal of the eye necessary, either to relieve pain or to prevent loss of sight in the other eye.

Although the question regarding the propriety of removing the globe can seldom give rise to doubts in the minds of an experienced practitioner, there are rare instances when the exercise of the most deliberate and wise judgment is requisite.

In general terms, it may be stated that the injuries which most frequently necessitate the removal of the globe are: 1st, foreign bodies within the intraocular tissues; 2d, concussions; and 3d, incisions, especially near the ciliary region.

In general terms, it may also be stated, that an eye should be extirpated, whenever, after an injury, the inflammation, especially of the iris and choroid fails to improve after a reasonable time, and there continues a tenderness on gentle pressure near the corneal border, with loss of vision.

*Foreign bodies in the globe.*—Of the specimens before you, 13 represent cases in which foreign bodies were lodged within the globe. In three of these cases, pus had formed in the vitreous humor, after a period varying from three days to three weeks. In four cases there was atrophy of all the ocular tissues, so that the sclerotic was deeply fissured in the region of the four recti muscles, and firmly contracted upon quite a hard mass of atrophied vitreous, choroid and retina. In six cases you perceive excellent examples of every stage of detachment of the retina; in some the retina resembles an umbrella partially closed; in two cases it has been pressed into the central portion of the globe, presenting the appearance of a cord passing from the optic nerve to the anterior part of the eye. (See Fig. 5.)

Fig. 5, a section of the eyeball, showing the "corded retina," from the effects of a foreign body.

*a.* The retina, folded into a dense round cord, by exudation between it and the choroid.

*b.* The position of the condensed and degenerated vitreous humor.

*c.* The foreign body (a small bird shot).

*d.* The choroid.

The substances which caused the destructive inflammation in these specimens, are fragments of steel, lead, percussion caps, and of wood. In none of the cases could their position be determined by a careful inspection with the ophthalmoscope, since the products of inflammation or hemorrhage concealed them.

It is worthy of remark that it is a most difficult manœuvre to remove a foreign substance from the vitreous humor, even when it can be seen through the pupil.

In three of these cases the patients had already lost the vision of the uninjured eye by sympathetic inflammation at the time of the extirpation.

*Concussions.*—In eight cases the globe was extirpated on account of injuries received by blows.

In two cases there is very great atrophy of all the tissues of the globe, with quite a large mass of calcareous deposit in place of the choroid.

In six others the globe has retained its normal size, but is characterized by total liquefaction of the vitreous. One case is especially worthy of notice, on account of the very hard coagulum between the sclerotic and choroid, which almost absolutely fills the whole globe, the retina and choroid being crowded to the opposite side. The patient had been in great pain for three months after receiving the blow.

*Incisions and Punctures.*—Of the twenty-eight specimens, eight illustrate the effects of this class of injuries. Almost without exception the wounds were quite large, and involved the ciliary region. They present considerable variety in the condition of the intra-ocular tissues; some are filled with a serous fluid, surrounded with a very thin choroid and retina, the iris being in contact with the cornea, and the ciliary bodies much attenuated. Others are less than normal in size, the internal membranes being thickened and indurated. In one specimen there is a remarkable thickness of the choroid and retina, each measuring nearly a sixteenth of an inch.

Two of these patients came under my care blind also in the uninjured eye from sympathetic inflammation.

As a rule of great practical importance, I repeat, that an eye which has been injured in any of the ways above mentioned, should be extirpated when vision has been destroyed, on account of irido-choroiditis, and, in spite of all treatment, the globe remains persistently tender on pressure over the anterior portion of the sclerotic.

I think I can except those cases, not numerous, however, in which, very early after the injury there is extensive suppuration of all the tissues of the globe, as indicated by great pain and extensive œdema of the ocular conjunctiva and of the lids. In these cases relief is secured by making an incision through the walls of the globe from the internal to the external angle of the palpebral fissure, and evacuating all the intra-ocular contents, including the choroid and retina. An artificial eye can be worn over the small "stump" somewhat more comfortably than when the whole globe is removed.

## Figures and Tables

**Fig. 5 f1:**